# The First Serological Detection and Risk Factors Analysis of Akabane Virus in Egyptian Cattle

**DOI:** 10.3390/ani13111849

**Published:** 2023-06-01

**Authors:** Samy Metwally, Nabil Bkear, Marwa Samir, Rania Hamada, Besheer Elshafey, Gaber Batiha, Taghreed N. Almanaa, Kamel Sobhy, Yassien Badr

**Affiliations:** 1 Division of Infectious Diseases, Department of Animal Medicine, Faculty of Veterinary Medicine, Damanhour University, Damanhour 22511, Egypt; nabil.baker@vetmed.dmu.edu.eg; 2 Laboratory of Global Infectious Diseases Control Science, Graduate School of Agricultural and Life Sciences, The University of Tokyo, Bunkyo-Ku, Tokyo 113-8657, Japan; 3 Faculty of Veterinary Medicine, Damanhour University, Damanhour 22511, Egypt; marrrw5@gmail.com; 4 Division of Clinical Pathology, Department of Pathology, Faculty of Veterinary Medicine, Damanhour University, Damanhour 22511, Egypt; rania.hamada@vetmed.dmu.edu.eg; 5 Division of Internal Medicine, Department of Animal Medicine, Faculty of Veterinary Medicine, Damanhour University, Damanhour 22511, Egypt; besheer_elshafey@vetmed.dmu.edu.eg; 6 Department of Pharmacology and Therapeutics, Faculty of Veterinary Medicine, Damanhour University, Damanhour 22511, Egypt; gaber_saber@dmu.edu.eg; 7 Department of Botany and Microbiology, College of Science, King Saud University, Riyadh 11451, Saudi Arabia; talmanaa@ksu.edu.sa; 8 Department of Theriogenology, Faculty of Veterinary Medicine, Damanhour University, Damanhour 22511, Egypt; kamelabozeid89@gmail.com

**Keywords:** Akabane disease, seroprevalence, ELISA, cattle, Egypt

## Abstract

**Simple Summary:**

Akabane virus (AKAV) is the etiologic agent of Akabane disease (AD), which causes severe economic losses among domestic and wild animals. In Egypt, AD has never been reported. Therefore, this study is the first to detect AKAV among Egyptian cattle. Out of 368 tested plasma samples obtained from cattle in Beheira province, north Egypt, the overall AKAV seroprevalence was 54.3% (95% CI: 50.8–61.4). Moreover, AKAV antibodies were detected in all examined cattle farms (7/7) and the majority of abattoirs (8/9). Age, sex, breed, and location of the tested cattle were analyzed as potential risk factors for AKAV infection. Meanwhile, age and breed were ultimately proven to be the most important risk factors.

**Abstract:**

Akabane virus (AKAV) is an insect-borne virus belonging to the genus *Orthobunyavirus* of the family *Peribunyaviridae.* It is the etiologic agent of Akabane disease (AD), which emerged in Asia, Australia, and the Middle East causing severe economic losses among domestic and wild animals. AKAV has not received enough attention in Egypt, and its evidence among Egyptian animals has never been reported. Therefore, this study used ELISA assay to investigate the seroprevalence of AKAV among Egyptian dairy and beef cattle in eight localities of Beheira province, north Egypt. Out of 368 investigated plasma samples, the overall AKAV seroprevalence was 54.3% (95% CI: 50.8–61.4). AKAV antibodies were detected in all examined cattle farms (7/7) and the majority of abattoirs (8/9). Age, sex, breed, and location of the tested cattle were analyzed as risk factors for AKAV infection. A higher significant increase in seropositivity was obtained in cattle who were aged >5 years (*p* < 0.0001; OR = 9.4), females (*p* < 0.0001, OR = 8.3), or Holstein breed (*p* < 0.0001, OR = 22.6) than in younger ages, males, and Mixed and Colombian zebu breeds, respectively. Moreover, a significant variation in AKAV seroprevalence between the tested locations was noticed. Ultimately, a multivariable analysis concluded that age (*p* = 0.002, OR = 3.32, 95% CI = 1.57–7.04) and breed (*p* = 0.03, OR = 1.69, 95% CI = 1.05–2.72) were significant risks for AKAV infection. In conclusion, this study is the first to detect AKAV infection in Egyptian animals.

## 1. Introduction

The Akabane virus (AKAV) is an arthropod-transmitted virus that is a member of the *Peribunyaviridae* family and the Simbu serogroup of the genus *Orthobunyavirus* (order *Bunyavirales*) [1]. It is the causative agent of the enzootic arthrogryposis-hydranencephaly condition known as Akabane disease (AD), which affects cattle, sheep, and goats [2]. The name AD has been used to characterize the clinical state brought on by an AKAV infection in utero since the virus was discovered and initially identified in Japan in 1959 [3,4]. The primary vectors of AKAV transmission are biting Culicoides species of midges. Despite the fact that AKAV has been isolated from mosquitoes, these insects are not thought to be the virus’s primary vectors [5,6]. AKAV infection in adult cattle is usually asymptomatic after a short period of transient fever. However, some AKAV strains can cross the placenta of pregnant cows and induce abortion and several abnormalities in the growing fetus [3,4]. These congenital defects vary according to the stage of pregnancy and include premature births, stillbirths, and other abnormalities of the CNS, such as porencephaly and microcephaly [5,6]. AD has resulted in severe economic losses in some nations. In Japan, this virus caused the birth of more than 42,000 deformed calves between 1972 and 1975 [7]. The primary economic losses linked to AKAV infection in dairy farms are infertility, early pregnancy abortions, dystocia during birth, and a significant decrease in the milk production of infected cows [8,9].

The AKAV genome is a single-stranded negative-sense RNA that is divided into three segments, the short (S), medium (M), and large (L), depending on their relative size [1,10]. A non-structural protein and the nucleocapsid protein are encoded by the S RNA segment. The M RNA segment codes for a polyprotein precursor that is post-translationally cleaved into the viral surface glycoproteins (Gn and Gc) and a non-structural protein (NSm). An RNA-dependent RNA polymerase is encoded by a single, big, open-reading frame found in the L RNA segment. Different roles for the encoded proteins are played in the viral life cycle, antigenicity, and neutralizing antibodies of the host defense [2,11,12]. AKAVs have been categorized into four separate groups (genogroups I–IV) based on genetic investigations, with genogroup I being further divided into two subgroups (Ia and Ib) [11,13]. Genogroups I and II have been assigned to all strains isolated from East Asia, which clearly distinguishes them from isolates from Australia and Africa that fall under genogroups III and IV [2].

AD has emerged as a common infection among many domestic and wild animals in many Asian nations, including Japan [14], South Korea [15], Taiwan [16], Iraq [17], and China, where it is one of the seven pandemic diseases that are subject to a mandatory quarantine, and prohibits the importation of cattle, sheep, and goats [18]. It has been also reported in a number of countries in the Middle East [19], and Australia [20]. In African countries, AKAV has been reported among free-living wild animals based on virus isolation, molecular methods, and serology [21], as well as in domestic animals in Sudan [9], Tanzania [22], Kenya [23], and Nigeria [24]. In addition to animals, AKAV has been reported in mosquito and Culicoides vectors [25].

Wide varieties of diagnostic techniques have been established for the diagnosis of AD in different animal species. Detection of AKAV-specific antibodies using serological methods is the most commonly used approach to AD diagnosis, such as a viral neutralization test, enzyme-linked immunosorbent assays (ELISAs) [26,27], dot immunobinding assay [28], and immunohistochemistry staining [29]. Successful molecular methods for the identification of the AKAV genome were developed using polymerase chain reaction (PCR), such as nested reverse transcription PCR (RT-PCR) [30], real-time RT-PCR [31], multiplex RT-PCR [32], and reverse transcription loop-mediated isothermal amplification assay (RT-LAMP) [33].

AKAV infection is a seasonal condition and its control measures are commonly related to insect vector activity [17]. Insect control in the breeding sites of animals and changing the calving seasons can be applied to prevent AKAV outbreaks [6]. Additionally, the use of vaccines for susceptible animals prior to the vector activity and the emergency vaccination of pregnant animals are strategic control methods [7].

Although AD has been recorded in neighboring countries, the presence of AKAV in Egyptian animals has never been recorded yet. Therefore, this study aimed to determine the seroprevalence of AD among cattle in Beheira province which comprises approximately 20% of the total cattle population in Egypt. Additionally, the influence of some risk factors such as age, sex, breed, and location on AKAV infection was analyzed.

## 2. Materials and Methods

### 2.1. Ethical Statement

The Institutional Committee of Ethics of the Faculty of Veterinary Medicine at Damanhour University, Egypt, has approved this research (DMU/VetINF-2022-/0154). Each owner provided their oral consent, as agreed upon by the Ethics Committee. All animals were handled in accordance with the Ethics Committee’s regulations and standards for animal care.

### 2.2. Farms and Animals

**Figure 1:**
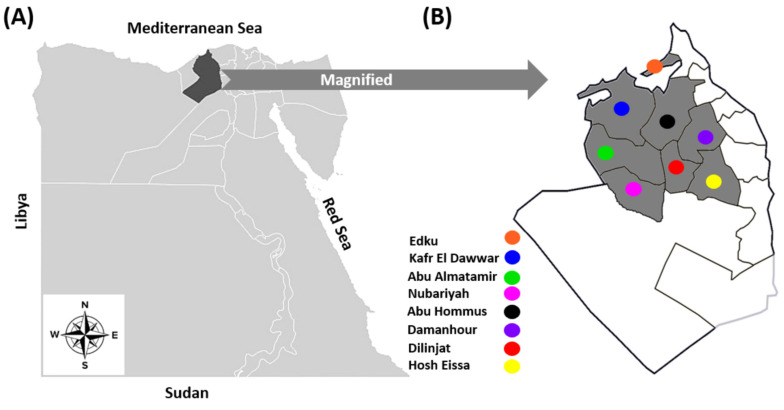
(**A**) The geographical map of Egypt shows the location of Beheira province in the northern region, indicated by the black landscape. (**B**) Magnified geographical map of Beheira province showing the eight districts where the samples were collected.

During an inclusive research project screening silent infections among cattle of Beheira province in 2022, a total of 368 cattle were randomly selected from either seven dairy and beef farms with a total of 4375 cattle heads or sporadic samples from nine abattoirs (Table 1). These cattle were distributed in eight different districts of Beheira province, north Egypt (Figure 1), based on the owner’s permission and without prejudice to any of the tested districts. Since the bovine sector in Egypt comprises more than 5 million heads and is directly correlated to the crops and cultivated areas, and Beheira province is the main center for crops and cereals production, thus it has the highest number of cattle farms in Egypt with approximately 20% of the total cattle population (i.e., more than one million cattle head) [34]. Cattle under investigation were classified according to the location: Damanhour (*n* = 20), Edku (*n* = 22), Abu Hommus (*n* = 83), Nubariyah (*n* = 57), Abu Almatamer (*n* = 27), Dilinjat (*n* = 60), Kafr El-Dawar (*n* = 76), and Housh Eissa (*n* = 23); age: <3 years (*n* = 149), 3–5 years (*n* = 143), and >5 years (*n* = 76); sex: males (*n* = 149), and females (*n* = 219); and breed: Mixed (*n* = 210), Holstein (*n* = 110), and Colombian zebu (*n* = 48). Notably, one to three farms and/or abattoirs from each locality were included, and on each farm, 10% of the animals were randomly selected for investigation, with the exception of an intensive dairy farm in Nubariyah. The selected animals appeared to be in good health, and none of them had any obvious signs of illness. Importantly, vaccination against AD is not carried out in Egypt.

### 2.3. Blood Sampling and Plasma Separation

A glass tube containing K2 EDTA anticoagulant was used to collect whole blood samples that were obtained from the cattle following the puncture of their tail veins. Blood samples were submitted quickly into the lab and kept at 4 °C. Plasma was separated for serological studies by centrifugation at 3000 rpm for 15 min at room temperature and then stored at −20 °C until analysis.

### 2.4. Serological Detection of Specific Anti-Akabane Antibodies by ELISA

Using a commercial ELISA kit, an indirect ELISA assay was conducted to serologically examine each of the 368 obtained cattle plasma samples for infection with AKAV. For the detection of specific anti-G1 antibodies of AKAV in the plasma samples, the steps of competitive ELISA specific for ruminants were followed according to manufacturer’s instructions (ID.vet, Grabels, France). The used ELISA kit was highly sensitive to AKAV antibodies in both plasma and serum samples equally, as specified in the instruction guide established by the manufacturer. The manufacturer also indicated that the kit had a high correlation (96.52%) with the virus neutralization test (VNT), which was confirmed by Li and colleagues, who found a 93.5% correlation [27] and had no cross-reactivity with other viruses in the *Bunyaviridae* family, such as Schmallenberg virus (SBV), Rift Valley fever virus (RVFV), and Aino virus. All ELISA results’ ODs were determined using an ELISA plate reader (Byonoy, Hamburg, Germany) and read at 450 nm. For each of the test samples, the measured optical densities (ODs) were used to compute the sample (S) to negative (N) ratio (S/N%) using the following formula: S/N (%) = OD sample/OD negative control × 100. Samples with an S/N% of more than 40% were considered negative, those with an S/N% between 30% and 40% were considered doubtful, and samples were considered positive if the S/N% was less than 30%.

### 2.5. Statistical Analysis

The seroprevalence of AKAV in the examined animals was calculated by counting. Statistical analysis was performed using SPSS Version 25 (IBM, New York, NY, USA). Regarding the univariable analysis of the risk factors associated with AKAV infection, the Fisher exact probability test (two-tailed) was used to estimate the significance of the differences in seroprevalence and risk factors. Additionally, the odds ratios (ORs) and 95% confidence intervals (95% CI) were calculated. Moreover, a multivariable logistic regression analysis was performed to identify the most important risk factor (s) (age, sex, breed, and location) associated with AKAV seropositivity. The *p*-values for entry into or removal from the logistic regression models were <0.05. The fit of the multivariable logistic regression model was estimated using the Hosmer–Lemeshow goodness-of-fit test, and then the confidence intervals for the odds ratio were obtained as described previously [35]. A *p*-value of <0.05 was considered statistically significant. An OR greater than 1 indicates an increased risk of the outcome (AKAV seropositive). Meanwhile, an OR less than 1 indicates a decreased risk of the outcome (AKAV seropositive) [35].

## 3. Results

In total, out of the 368 tested plasma samples, 200 (54.3%, 95% CI: 50.8–61.4) were positive for AKAV antibodies (Table 2). On the districts level, the AKAV seroprevalence rates were 25.0%, 86.4%, 45.8%, 70.2%, 88.9%, 51.7%, 53.9%, and 8.7% in Damanhour, Edku, Abu Hommus, Nubariyah, Abu Almatamer, Dilinjat, Kafr El-Dawar, and Housh Eissa, respectively (Table 2).

On the farm and abattoir levels, seropositive samples were detected in 15 of 16 investigated farms and abattoirs. However, sporadic samples from abattoir #9 that handled Colombian zebu cattle were totally seronegative (Table 3). In dairy farms, a higher seroprevalence of AKAV was reported in the farms comprising Mixed breeds, with percentages of 86.4%, 88.9%, 65.2%, and 74.0% in farms #1, 4, 5, and 7, respectively, than 67.5%, and 70.2% in farms #2 and 3 that maintained Holstein cows, respectively. Regarding the beef cattle farm, the serological detection of AKAV in beef farm #6, which maintained Mixed and Holstein breeds, indicated the highest seroprevalence (51.9%) among the tested beef cattle. Furthermore, the sporadic samples from abattoirs that included either Mixed or Mixed and Colombian zebu beef cattle showed seroprevalences of 26.7%, 17.0%, 35.0%,18.7%, and 14.3% for abattoirs #1, 3, 4, 6, and 8, respectively. However, seroprevalences of 20.0%, 20.0%, 10.0%, and 0.0% were detected in the tested Colombian zebu cattle obtained from abattoirs #2, 5, 7, and 9, respectively.

The influence of some risk factors, such as age, sex, breed, and location of the tested cattle, on the seropositivity of AKAV was investigated using a univariable analysis (Table 4). Regarding the effect of age on AKAV infection, the seroprevalence in cattle aged <3 years old was 38/149 (25.5%), while a significant increase (*p* < 0.0001; OR = 7.8) in seropositivity was reported in cattle aged 3–5 years (104/143; 72.7%). However, the highest significance (*p* < 0.0001; OR = 9.4) in seropositivity was demonstrated in older cattle aged >5 years (58/76, 76.3%) (Table 4). Female cattle showed a significantly higher seroprevalence of AKAV (162/219, 74%; *p* < 0.0001, OR = 8.3) than males (38/149, 25.5%; Table 4). Notably, based on the production type of the tested animals, all dairy cattle were females, whereas all beef cattle were males (Table 3). Regarding cattle breeds, significant differences in the seropositivity for AKAV between Mixed, Holstein, and Colombian zebu animals were recorded. The lowest seroprevalence of AKAV infection was detected in Colombian zebu (4/48; 8.3%). Mixed cattle showed a higher (122/210; 58.1%), significant (*p* < 0.0001, OR = 15.3) seroprevalence, while the Holstein cattle breed showed the highest (74/110; 67.3%), significant (*p* < 0.0001, OR = 22.6) infection rate among the tested breeds (Table 4).

The seroprevalence of AKAV in the eight investigated districts were greatly variable. Seroprevalence of AKAV in the capital city of Beheira province, namely Damanhour (5/20; 25%), was taken as a reference value, and thus cattle in Edku (19/22; 86.4%), Nubariyah (40/57; 70.2%), Abu Almatamer (24/27; 88.9%), Dilinjat (31/60; 51.7%), and Kafr El-Dawar (41/76; 53.9%) had significantly higher values in comparison (*p* = 0.0001, 0.0006, <0.0001, 0.04, 0.025; ORs = 19, 7.1, 24, 3.2, 3.5, respectively). A non-significant increase (*p* = 0.13, OR = 2.5) in AKAV seroprevalence was reported in Abu Hommus (38/83; 45.8%) compared to Damanhour. However, a non-significant decrease (*p* = 0.22, OR = 0.3) in AKAV seropositivity was observed in Housh Eissa (2/23; 8.7%) (Table 4).

The multivariable logistic regression analysis for the different variables associated with AKAV seroprevalence in cattle tested indicated that age (*p* = 0.002, OR = 3.32, 95% CI = 1.57–7.04) and breed (*p* = 0.03, OR = 1.69, 95% CI = 1.05–2.72) were significant risk factors for AKAV infection (Table 5). However, sex (*p* = 0.74, OR = 1.22, 95% CI = 0.38–3.89) and location (*p* = 0.49, OR = 0.96, 95% CI = 0.85–1.08) of the cattle tested were not associated with a greater risk for AKAV seropositivity (Table 5).

## 4. Discussion

AD is commonly occurring in most tropical and subtropical regions between latitudes 35° N and 35° S because of the distribution of the insect vector [6]. Although Egypt is situated in the subtropical climatic zone between latitudes 22° N and 32° N, where the climatic conditions are favorable for the insect vector’s habitat, AKAV has not received enough attention as a potential cause of arthrogryposis-hydranencephaly syndrome and its evidence among Egyptian animals is still poorly identified. Therefore, this study firstly focused on the investigation of infection with AKAV among Egyptian cattle, particularly in Beheira province, where animal farming is the most important sector due to the agriculture of wheat, rice, corn, potatoes, and sugar beets, which are required for animal nutrition [36]. Secondly, it aimed to determine the effect of potential risk factors of sex, age, breed, and location of the investigated animals on the seroprevalence of AKAV. The main findings in this study revealed the endemic situation of AKAV in tested cattle, and that overall, age (*p* = 0.002, OR = 3.32) and breed (*p* = 0.03, OR = 1.69) were the biggest potential risks influencing AKAV seropositivity.

The total seroprevalence of AKAV antibodies among the investigated cattle was 54.3% (95% CI: 50.8–61.4), as determined serologically by ELISA kits. Since AKAV vaccines are not currently given to livestock in Egypt, the discovery of seropositive cattle in this study shows that these animals were exposed to AKAV naturally. The obtained seroprevalence of AKAV antibodies in Egyptian cattle was high and relatively higher than the seroprevalence using the same ELISA kits in Sudan, with an overall prevalence rate of 29.4% and the prevalence rates of AKAV antibodies in Sudanese cattle varying between 69.6% in Khartoum state and 3.3% in Sennar State [9]. A previous study by Mohamed et al. [37] that assessed the prevalence of AKAV antibodies in domestic ruminants in Sudan found that 47% of cattle had these antibodies. In contrast, a higher AKAV seropositivity (70.1%) was reported in Nigerian cattle [24]. Furthermore, an evaluation of 42 newborn calves in Iraq with hydranencephaly and arthrogryposis indicated 100% seropositivity for AKAV antibodies [17]. These nations, including Egypt, share similar climatic conditions that contribute to the spread of AKAV infections. The climatic conditions and range of insect vector populations are likely to have traditionally influenced how the AKAV spreads [38].

It is interesting to note, however, that despite the high obtained AKAV seroprevalence among tested cattle in Egypt, no epidemics of congenital abnormalities in such cattle have been documented there, despite reports to the contrary in other areas of the world. For instance, neurological diseases epizootics caused 500 deaths in South Korea in 2010 and about 180 deaths in Japan in 2006 [13,39] and there have also been reports of congenital arthrogryposis-hydranencephaly syndrome in calves [7,17]. This might be because there have not been any studies linking AKAV infection to deformities in newborn calves, or it might be because there are different unknown strains of the virus circulating in Egypt. Another reason might be that AKAV infections in cattle are typically asymptomatic and only transient fever has typically been detected in animals without any other obvious clinical signs [21].

Notably, the findings showed that AD was widely distributed in all of the investigated farms and the majority of abattoirs (15/16 seropositive). AKAV seropositivity was higher in all seven tested farms (dairy and beef), where seroprevalence ranged from 51.9% to 88.9%, compared to sporadic samples in all nine abattoirs, where seroprevalence ranged from 0.0% to 35.0%. In contrast, Nigerian researchers found that cattle farms had a seroprevalence of 40.0%, while abattoirs had a seroprevalence of 73.8% [24]. The variation in seroprevalence between dairy and beef cattle might be because beef and dairy farming systems in Egypt operate differently, either intensively (more than 200 cattle per farm), semi-intensively (20–200 cattle per farm), or smallholders (5–20 cattle per farm) [40]. Based on this classification of farms in this study, it was surprising that the seroprevalence of AKAV in semi-intensive farms, such as farms #1 and 4, was absolutely higher than in intensive farms, such as farms #2, 3, 5, and 7. The lower seropositivity in intensive farms is possibly due to the management practice in this farming system [34], or might be affected by other factors such as the breed of tested cattle [9]. Conversely, intensive farming practices commonly used for dairy cows may contribute to AKAV’s spread via insect vectors, as confirmed previously by Elhassan et al. [9], who noted a higher seroprevalence (37%) in animals under intensive management than in animals under the extensive system (11.0%). However, we could not analyze the risk of AKAV infection in beef and dairy cattle based on the farm size because most of the beef cattle tested originated from sporadic samples in abattoirs with unknown farm sizes.

Age, sex, breed, and location of cattle under investigation were considered potential risk factors for AKAV infection. Regarding age, compared to younger (25.5%) cattle, older (76.3%) and mid-aged (72.7%) animals showed significantly higher seropositivity. This might be because older animals were exposed to the AKAV infection by insect vectors for longer periods of time than young calves. In agreement with our results, Oluwayelu et al. [24] reported higher seropositivity in adult cattle (86.4%) than in younger (14.3%). On the contrary, Elhassan et al. [9] obtained a higher increase in mid-aged cattle (45.3%) than adults (34.8%). Collectively, females represented the majority of older cattle that were kept for dairy production and showed a higher seroprevalence (74%) than males (25.5%) that were kept for beef production and aged under three years. In agreement with the results of a recent study in Sudan, the prevalence of AKAV was higher in females (33%) than in males (14.3%) [9]. It would be thought that dairy herds which frequently breed females are commonly suitable environments for the habitat of insect vectors [9,41].

Considering breeds of tested animals, three breeds of cattle were investigated. The Colombian zebu cattle that were imported from Colombia, South America had the lowest seroprevalence (8.3%); thus, higher seropositivity was detected in imported Holstein (67.3%), and Mixed cattle (58.1%) in comparison. The demonstrated wide variation in seropositivity among foreign breeds might be directly linked to the period of stay of such breeds in Egypt, age, or the farming system of cattle tested. Notably, Egypt imports beef breeds (e.g., Colombian zebu) from South American countries for either immediate slaughter or to be kept for several months for fattening in beef farms. On the other side, dairy heifers of highly milk-producing breeds (e.g., Holstein) that are usually imported from European countries and the United States are reared for lifelong breeding in intensive dairy farms [42]. To our knowledge, there were no previous studies that demonstrated that Colombian zebu cattle differ from other cattle breeds in their susceptibility to pathogens. However, the low AKAV seroprevalence in Colombian zebu cattle may be affected by the distribution of the virus; that is, none of the Simbu serogroup viruses that infect animals have been found in South America, despite the fact that some of them that cause febrile illnesses in humans have been found in the Caribbean and South America [6]. Although the AKAV is not common in European countries and the United States [6], it was noticed that Holstein cattle exhibited a greater (67.3%) prevalence than Mixed cattle (58.1%). In Egypt, a Mixed breed is a crossbreed resulting from the breeding of Egyptian native and foreign cattle and is usually kept in smallholder or semi-intensive farms individually or with other cattle breeds [43]. Therefore, it is expected that rearing Mixed and Holstein breeds together under Egyptian farming conditions could increase the possibility of AKAV transmission to each other. For example, the small beef farm #6 which kept both breeds showed the highest seroprevalence (51.9%) among all beef cattle tested. However, this was the only farm of its type, which influenced the increase in the seroprevalence of Holstein cattle. In this context, crossbreeds were found to be a risk factor for AKAV transmission to native cattle in Sudan [9]. Ultimately, the difference in seroprevalence between breeds seems to be related to the area from which the cattle were imported and the period during which they were kept in Egypt. That is, because Colombian zebu cattle are imported from an Akabane disease-free area and are kept in Egypt for a short period (i.e., several months), they have a lower chance of being infected with AKAV compared to other breeds.

Although the investigated districts were located geographically near each other and under the same climatic conditions, the seroprevalence of AKAV greatly varied between the districts tested. Despite following a random sampling strategy, this variation in seropositivity is possibly affected by the production type, breed, or farm size of the cattle tested. For example, the lowest seroprevalence was reported in Housh Eissa (8.7%) and Damanhour (25%) districts, where all tested animals were beef cattle that were categorized as sporadic samples of either Mixed or Colombian zebu breeds submitted for slaughter in abattoirs. Contrary, the highest seroprevalence of AKAV was estimated in Abu Almatamer (88.9%), Edku (86.4%), and Nubariyah (70.2%) districts, where all tested animals were dairy cattle of either Mixed or Holstein breeds obtained from farms with variable sizes. The endemic situation of AKAV in all districts tested might cause severe economic losses and predict the occurrence of future outbreaks in Egypt, as previously happened in Japan [7], Republic of Korea [39], and Iraq [17]. Therefore, we recommend the application of insect control measures during the vector activity and alternative management practices in the Egyptian farms such as changing the calving period from spring to autumn [6]. Additionally, a study disclosing the current economic impact of AKAV infection on Egyptian cattle is needed.

The present study had two limitations that should be mentioned. First, although the subject of this study is the prevalence of antibodies against AKAV in Egyptian animals, where the disease has never been reported before, it was carried out with a small number of samples from a single Egyptian province. Therefore, a comprehensive national-wide study of AKAV infection across various Egyptian provinces could shed more light on the disease’s epidemiology. Second, despite the use of a highly sensitive and specific ELISA kit as an individual serological test in numerous previous studies for AKAV detection [9,17,24,27], we believe that additional comparative diagnostic methods for AKAV prevalence and its molecular characterization in Egyptian livestock are required.

## 5. Conclusions

The findings in this study presented the first report of AKAV in Egyptian cattle by serological methods using ELISA kits. The overall seroprevalence was high (54.3%) and relatively higher than the neighboring African countries. Statistical analysis of the variables indicated the potential impact of age, sex, breed, and location of the investigated cattle on AKAV seroprevalence. Age and breed were ultimately proven to be the most important risk factors. Moreover, future molecular detection and characterization of the circulating AKAV strains in cattle in Egypt are needed. Additionally, the improvement of diagnostic techniques in laboratories to detect AKAV and the reporting systems for the clinical signs of AD such as abortion and congenital malformations among Egyptian animals are urgently required.

## Figures and Tables

**Table 1:** Number and locations of tested samples of cattle from Beheira, Egypt.

	Location	Damanhour	Edku	Abu Hommus	Nubariyah	Abu Almatamer	Dilinjat	Kafr El-Dawar	Housh Eissa	Total
Population										
No. of tested farms/abattoirs		0/2	1/0	1/2	1/0	1/0	2/1	1/2	0/2	7/9
No. of samples from farms/abattoirs		0/20	22/0	40/43	57/0	27/0	23/37	50/26	0/23	368
Farm size (Cattle per farm) *		-	195 ^b^	750 ^a^	2000 ^a^	100 ^b^	630 ^a^	700 ^a^	-	4375

^a^ Intensive farms (>200 heads per farm), ^b^ semi-intensive farms (50–200 heads per farm); * cattle from abattoirs were sporadic samples of unknown farm sizes; (-) means cattle in such area were obtained from abattoirs only.

**Table 2:** Seroprevalence of Akabane virus antibodies in tested cattle of Beheira.

Location	No. of Tested	No. of Negative (%)	No. of Doubtful (%)	No. of Positive (%)	95% CI *
Damanhour	20	15 (75.0)	0 (0.0)	5 (25.0)	9.6–49.4
Edku	22	2 (9.1)	1 (4.5)	19 (86.4)	68.2–98.3
Abu Hommus	83	41 (49.4)	4 (4.8)	38 (45.8)	36.8–59.5
Nubariyah	57	17 (29.8)	0 (0.0)	40 (70.2)	56.4–81.2
Abu Almatamer	27	3 (11.1)	0 (0.0)	24 (88.9)	69.7–97.1
Dilinjat	60	26 (43.3)	3 (5.0)	31 (51.7)	40.8–67.4
Kafr El-Dawar	76	31 (40.8)	4 (5.3)	41 (53.9)	44.8–68.4
Housh Eissa	23	21 (91.3)	0 (0.0)	2 (8.7)	15.2–29.5
Total	368	156 (42.4)	12 (3.3)	200 (54.3)	50.8–61.4

* CI: confidence interval.

**Table 3:** Seroprevalence of Akabane virus antibodies among farm and abattoir levels.

Sample Origin	Region	Sex	Breed	Type of Production	No. of Tested	No. of Negative (%)	No. of Doubtful (%)	No. of Positive (%)	Seropositive Status	95% CI
Abattoir #1	Damanhour	Male	Mixed	Beef	15	11 (73.3)	0 (0.0)	4 (26.7)	Yes	8.9–55.2
Abattoir #2	Damanhour	Male	Colombian Zebu	Beef	5	4 (80)	0 (0.0)	1 (20)	Yes	1.1–70.1
Farm #1	Edku	Female	Mixed	Dairy	22	2 (9.1)	1 (4.5)	19 (86.4)	Yes	68.2–98.3
Farm #2	Abu Hommus	Female	Holstein	Dairy	40	10 (25.0)	3 (7.5)	27 (67.5)	Yes	55.6–85.6
Abattoir #3	Abu Hommus	Male	Mixed and Colombian Zebu	Beef	23	19 (82.6)	0 (0.0)	4 (17.4)	Yes	5.7–39.5
Abattoir #4	Abu Hommus	Male	Mixed and Colombian Zebu	Beef	20	12 (60.0)	1 (5.0)	7 (35.0)	Yes	17.2–61.3
Farm #3	Nubariyah	Female	Holstein	Dairy	57	17 (29.8)	0 (0.0)	40 (70.2)	Yes	56.4–81.2
Farm #4	Abu Almatamer	Female	Mixed	Dairy	27	3 (11.1)	0 (0.0)	24 (88.9)	Yes	69.7–97.1
Farm #5	Dilinjat	Female	Mixed	Dairy	23	7 (30.4)	1 (4.3)	15 (65.2)	Yes	45.1–85.3
Farm #6	Dilinjat	Male	Holstein and Mixed	Beef	27	11 (40.7)	2 (7.4)	14 (51.9)	Yes	35.3–75.0
Abattoir #5	Dilinjat	Male	Colombian Zebu	Beef	10	8 (80.0)	0 (0.0)	2 (20.0)	Yes	35.4–55.8
Farm #7	Kafr El-Dawar	Female	Mixed	Dairy	50	10 (20.0)	3 (6.0)	37 (74.0)	Yes	63.9–88.8
Abattoir #6	Kafr El-Dawar	Male	Mixed	Beef	16	12 (75.0)	1 (6.3)	3 (18.7)	Yes	5.3–48.6
Abattoir #7	Kafr El-Dawar	Male	Colombian Zebu	Beef	10	9 (90.0)	0 (0.0)	1 (10.0)	Yes	0–45.9
Abattoir #8	Housh Eissa	Male	Mixed	Beef	14	12 (85.7)	0 (0.0)	2 (14.3)	Yes	2.5–43.8
Abattoir #9	Housh Eissa	Male	Colombian Zebu	Beef	9	9 (100.0)	0 (0.0)	0 (0.0)	No	0–37.1
Total	8	2	3	2	368	156 (42.4)	12 (3.3)	200 (54.3)	15/16 positive	50.8–61.4

CI: confidence interval.

**Table 4:** Univariable analysis for risk factors associated with Akabane virus infection in cattle of Beheria, Egypt.

Analyzed Factor	No. of Tested	No. of Negative (%)	No. of Positive (%)	OR (95% CI) ^#^	*p*-Value *
**Age**					
<3 years	149	111 (74.5)	38 (25.5)	Ref.	Ref.
3–5 years	143	39 (27.3)	104 (72.7)	7.8 (4.6–13.1)	<0.0001
>5 years	76	18 (23.7)	58 (76.3)	9.4 (4.9–18.1)	<0.0001
**Sex**					
Male	149	111 (74.5)	38 (25.5)	Ref.	Ref.
Female	219	57 (26)	162 (74)	8.3 (5.1–13.2)	<0.0001
**Breed**					
Mixed	210	88 (41.9)	122 (58.1)	15.3 (5.5–40.6)	<0.0001
Holstein	110	36 (32.7)	74 (67.3)	22.6 (7.9–61.3)	<0.0001
Colombian zebu	48	44 (91.7)	4 (8.3)	Ref.	Ref.
**Location**					
Damanhour	20	15 (75)	5 (25)	Ref.	Ref.
Edku	22	3 (13.6)	19 (86.4)	19 (3.9–73.8)	0.0001
Abu Hommus	83	45 (54.2)	38 (45.8)	2.5 (0.9–6.8)	0.13
Nubariyah	57	17 (29.8)	40 (70.2)	7.1 (2.1–19.8)	0.0006
Abu Almatamer	27	3 (11.1)	24 (88.9)	24 (5.1–91.9)	<0.0001
Dilinjat	60	29 (48.3)	31 (51.7)	3.2 (1.0–8.7)	0.04
Kafr El-Dawar	76	35 (46.1)	41 (53.9)	3.5 (1.2–9.4)	0.025
Housh Eissa	23	21 (91.3)	2 (8.7)	0.3 (0.05–1.7)	0.22

^#^ Odds ratio at 95% confidence interval; * *p* value was evaluated by Fisher exact probability test (two-tailed). The result is significant at *p* < 0.05; Ref. is a value that was used as a reference.

**Table 5:** Multivariable analysis for risk factors associated with Akabane virus infection in cattle of Beheria, Egypt.

Variables	Estimated Value	SE	*p*-Value *	OR	95% CI_OR_	
					Lower	Upper
Constant	−3.19	0.62	0.0001			
Age	1.20	0.38	0.002	3.32	1.57	7.04
Sex	0.19	0.59	0.74	1.22	0.38	3.89
Breed	0.52	0.24	0.03	1.69	1.05	2.72
Location	−0.04	0.06	0.49	0.96	0.85	1.08

SE: standard error; OR: odds ratio; CI: confidence interval; * the result is significant at *p* < 0.05.

## Data Availability

All data generated or analyzed during this study are included in this article.
